# Leiomyosarcoma of the inferior vena cava: Clinical experience with four cases

**DOI:** 10.1186/1477-7819-4-1

**Published:** 2006-01-04

**Authors:** Said Abisi, Gareth J Morris-Stiff, David Scott-Coombes, Ian M Williams, Anthony G Douglas-Jones, Malcom C Puntis

**Affiliations:** 1 Department of Surgery, University Hospital of Wales, Heath Park, Cardiff, CF14 4XN, UK; 2 Department of Pathology, University Hospital of Wales, Heath Park, Cardiff, CF14 4XN, UK

## Abstract

**Background:**

Leiomyosarcoma of the inferior vena cava is a rare tumor that presents in an insidious manner with non-specific symptoms. Given its rarity, there are no consensus guidelines to its management. The aim of this study was to report the clinical experience in the management of patients presenting to our institution during a 12 year period.

**Patients and Methods:**

Four patients with leiomyosarcomas of the inferior vena cava were managed at our institution during the period reviewed. Patient details were identified through a search of the pathology department computerized database, and case notes were retrospectively reviewed to obtain details of presentation and management.

**Results:**

There were 3 females and 1 male with a mean age of 59 years. All tumors were identified within 2 months of first symptoms. Three of the 4 had localized tumors whilst 1 patient had lung metastases at presentation. The three patients with resectable tumors underwent radical surgical excision of the tumor, and two patients had postoperative radiotherapy. One patient died of recurrence at 7 months, and another at 30 months. The third patient is currently well and disease free at 16 months. The fourth patient with metastatic disease was treated with chemotherapy alone and survived 36 months.

**Conclusion:**

Leiomyosarcoma of the inferior vena cava is an uncommon tumor that presents with non-specific symptoms. At the time of presentation, tumors are usually large and resection is challenging but probably offers the best opportunity for long-term survival.

## Background

Leiomyosarcomas of the inferior vena cava (IVC) are rare as illustrated by the fact that an International IVC Leiomyosarcoma registry established in 1992 collected, by means of literature review and personal communication, only 218 cases by the time of publication of its first report in 1996 [1]. Given their anatomical location, it is not surprising that they often present insidiously with non-specific symptoms such as: abdominal pain, weight loss, mass, fever, weakness, anorexia, vomiting, night sweats, dyspnoea, or they may present with Budd-Chiari syndrome [2,3].

IVC Leiomyosarcomas are classified anatomically according to their relationship to the liver and renal vessels: lower or Segment I (below the renal vessels); middle or Segment II (renal vessels to retrohepatic IVC) and upper or Segment III (suprahepatic IVC) [4]. The management of these tumors has been, where possible, by means of radical *en-bloc *resection with a view of achieving clear margins. However, in spite of an overall 50% 5-year survival, half of the tumors recur either locally or metastasize [1,3].

We present our experience with four cases of IVC leiomyosarcoma managed at our institution between January 1993 and December 2004.

## Patients and methods

From January 1993 to December 2004, 4 patients were diagnosed with leiomyosarcomas of the IVC. There were 3 females and 1 male with a mean age of 59 years (range: 57–61 years). The demographic details and symptoms at presentation are summarized in Table 1.

**Table 1:** Demographic features and presenting symptoms of patients with IVC leiomyosarcoma.

	**Patients**			
	
	**1**	**2**	**3**	**4**
**Demographics**				
Sex	Female	Female	Male	Female
Age	57	60	57	61
**Symptoms & Signs**				
Abdominal Pain	Yes	Yes	No	Yes
Weakness	No	Yes	Yes	Yes
Anorexia	No	Yes	No	Yes
Vomiting	No	No	No	Yes
Night sweats	No	No	No	No
Dyspnoea	No	No	No	No
Mass	No	No	No	No
Fever	No	No	No	No
Abdominal wall veins distension	Yes	No	No	No
**Diagnosis**				
Modalities used	USS + CT	USS + CT	CT	USS + CT
Location	Lower	Middle	Upper	Middle
Size	8 × 14 cm	8 × 7 cm	10 × 10.5 cm	9.5 × 7 cm
Embolus present	No	No	Yes	No
Metastasis present	Yes (Lung)	No	No	No
Biopsy performed	Yes	No	No	No

USS – ultrasound scan; CT – computed tomography

**Table 2:** Management and outcome data for patients with IVC leiomyosarcoma.

	**Patients**			
	**1**	**2**	**3**	**4**

**Management**				
Operation	No	Resection and IVC PTFE Patch	Resection and primary repair	Resection and IVC Vein Patch
Chemotherapy	Yes	No	No	No
Adjuvant therapy	None	Radiotherapy	None	Radiotherapy
Histological grade	High	High	High	High
**Complications**				
Limb oedema	NA	No	No	No
Venous insufficiency	NA	No	No	No
Other	None	No	CVA	No
**Follow-up**				
Recurrence	-	No	Yes (5 months)	No
Status	Dead	Dead	Dead	Alive
Survival	36 months	30 months	7 months	16 months

NA – not applicable; CVA – cerebrovascular accident

Clinical data was obtained from review of the patient notes, whilst radiological and histological results were obtained from prospectively maintained departmental computerized databases.

## Results

All 4 patients were symptomatic at presentation although there was significant variation in symptoms between patients (Table 1). Patient 1 was referred specifically for investigation of distended abdominal wall veins.

**Figure 1:**
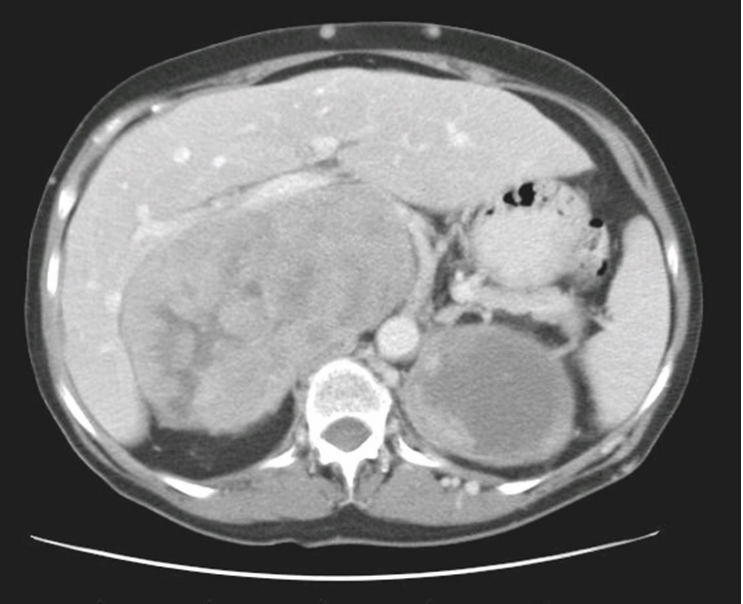
Computerized tomographic scan of patient 1 demonstrating a large tumor.

The diagnosis of a caval tumor was made on an ultrasound scan (USS) in 3 of the 4 cases and on computed tomography (CT) in the fourth. CT scans were performed to further characterize the origin of the tumors, demonstrate any contiguous invasion, and to exclude extra-abdominal metastases (Figure 1). Patient 3 was diagnosed with a tumor embolus to the right atrium preoperatively. No further preoperative imaging was performed. Given the location of the tumors, and based on experience with other sarcomas, no biopsy was performed preoperatively for the 3 patients undergoing surgical excision. Patient 1 underwent biopsy of the primary tumor confirm the diagnosis prior to commencement of chemotherapy.

Of the patients who underwent laparotomy, the tumor was excised *en-bloc *in all 3 cases. Patient 3 also underwent a thoracotomy and excision of a tumor embolus from the right atrium. In this patient, the point of origin of the tumor on the IVC was narrow and there was enough mobility to primarily close the IVC. Whilst in the remaining 2 cases, the tumor had a broad base. In these cases, the tumor was excised with a generous cuff of normal IVC wall, the defect was patched with a portion of long saphenous vein in patient 4 (Duplex ultrasound was used preoperatively to demonstrate the absence of deep venous thromboses, and deep venous incompetence in the lower limbs. In-patient 2 a PTFE patch was used to repair the IVC defect.

**Figure 2:**
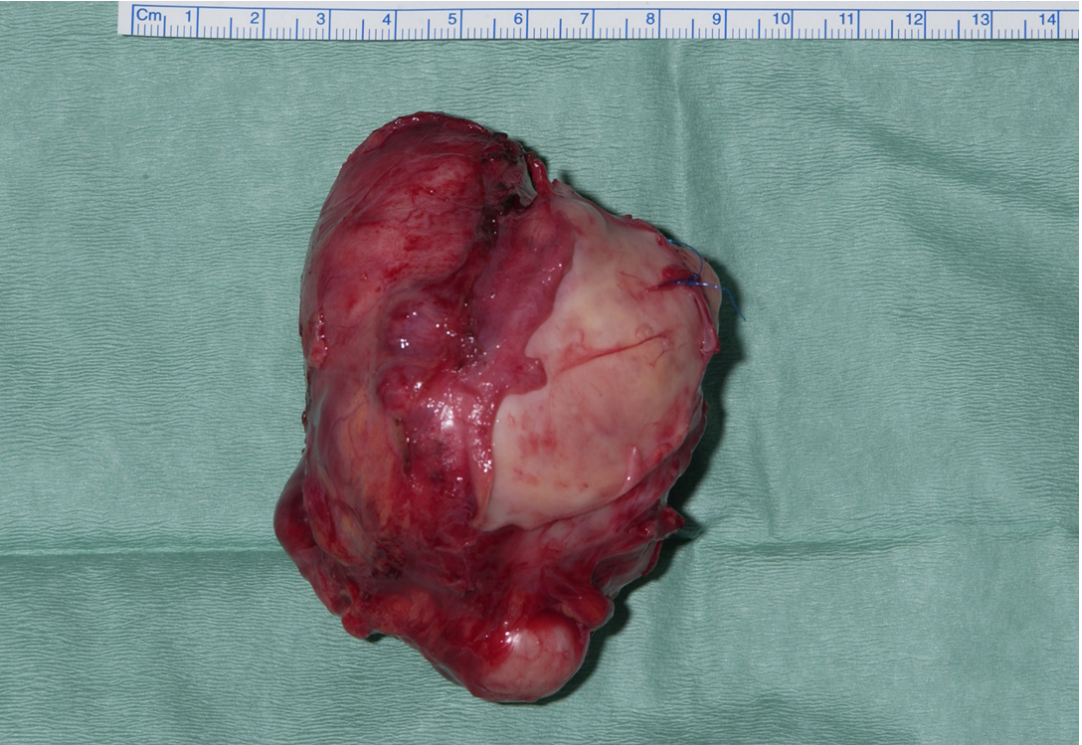
Gross appearance of the tumor.

**Figure 3:**
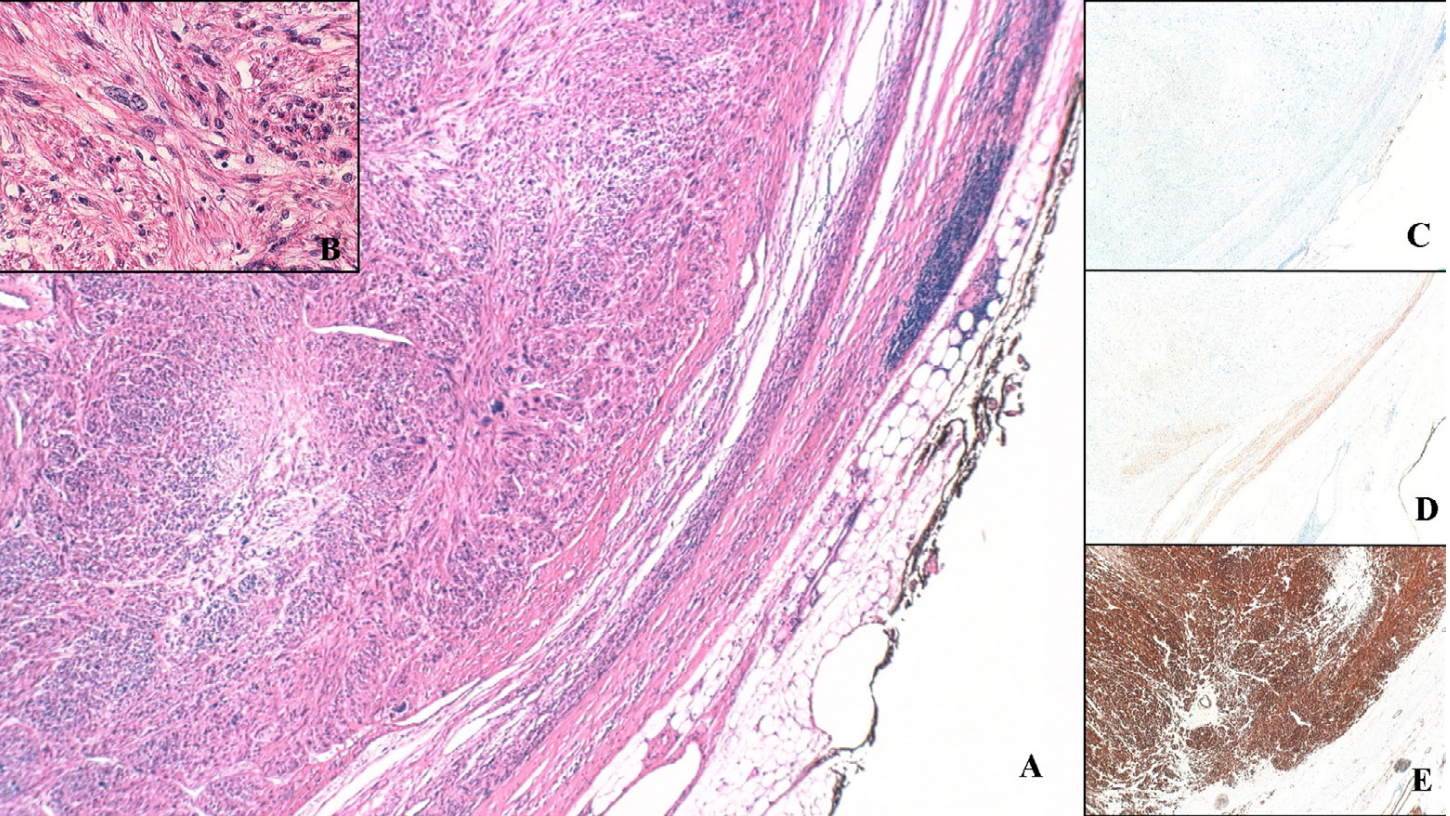
**Photomicrograph of the edge of the tumour (× 40). **(A). The tumour is composed of spindle cells which show nuclear pleomorphism with occasional giant nuclei (Hematoxylin and eosin × 200), (B). Immunohistochemistry for epithelial marker AE1/AE3 (C), and for S100 (D) is negative, but tumor is strongly positive for smooth muscle marker smooth muscle actin(E).

All tumors were large volume masses and had the classical tan-colored, nodular appearance (Figure 2). Histological examination revealed all to be high-grade leiomyosarcomas composed of spindle cells with positive immunohistochemistry staining for Desmin, HHF 35(muscle specific actin), SMA (smooth muscles actin), and negative for epithelial marker AE1/AE3, S100 protein confirming smooth muscles origin, and excluding nerve sheath origin (Figure 3).

The postoperative course was unremarkable for 2 patients. Patient 3, however, suffered a cerebrovascular accident perioperatively, but subsequently made a full recovery. Importantly, there were no complications in relation to the venous blood flow in the inferior vena cava and no renal impairment. Two of the patients (patient 2 and 4) undergoing resection received postoperative radiotherapy.

Patient 1 had a variety of chemotherapy protocols as well as palliative therapy, and survived 36 months. Disease recurrence occurred in patients 2 and 3. Patient 3 presented with widespread metastases and died at 7 months, whilst patient 2 developed disseminated disease 2 years after his surgery and died at 30 months post resection. Patient 4 is alive and disease free at 16 months post surgery.

## Discussion

The first reported case of leiomyosarcoma of the inferior vena cava was documented in the German literature by Perl [5] in 1871 but as of 1996 only 218 had been collected [1]. Although only 2% of leiomyosarcomas are vascular in origin, tumors of the IVC account for at least half the cases [4]. The majority of cases (75–90%) occur in women [4].

Leiomyosarcomas of the IVC are rare malignant tumors originating from the smooth muscle cells of the media of the cava and demonstrate three growth patterns – extraluminal, intraluminal or both [6]. The tumors are encapsulated, consisting of lobulated whorls, and histological examination demonstrates typical spindle-shaped bundles of cells with high mitotic activity and positive staining for desmin, HHF35, vimentin and smooth muscle actin.

This tumor commonly presents with a few months history of vague upper abdominal symptoms including pain, anorexia and vomiting; but may also present with more distinct signs such as an abdominal mass, deep venous thrombosis due to IVC occlusion, or less commonly as an acute Budd-Chiari Syndrome. However metastasis may be the first presentation of the tumor such as in case 1.

Given the rarity of the tumor, and in the absence of symptoms and signs of IVC compression, the identification of an IVC leiomyosarcoma is often an unexpected finding on imaging with USS or CT. In 3 of the 4 cases in this series, the tumor was identified on USS and a CT scan was performed to determine the origin, evaluate any local invasion of the tumor and exclude pulmonary metastases. In all 3 cases undergoing surgery, a diagnosis of leiomyosarcoma was suspected preoperatively. Other imaging modalities that aid the diagnosis of IVC sarcomas include vena cavography, which allows biopsy of the tumor. Nevertheless, in some cases, the precise origin of the tumor cannot be confirmed preoperatively. Furthermore, percutaneous biopsy to confirm histology of tumors in this region is often not possible due to the anatomical location of the IVC. Thus the surgeon is often faced with a tumor of uncertain type and origin at laparotomy.

Whilst there is no strong evidence base for the management of IVC leiomyosarcomas, the treatment of choice based on the available literature is radical *en-bloc *surgical excision with a view to obtaining negative resection margins. A number of techniques have been reported for dealing with the IVC following excision including IVC ligation; primary repair of IVC; patching of IVC and interposition grafting with a synthetic conduit. In the largest single institute series of 25 patients over a 20 year period, Hollenbeck *et al *[7], concluded that primary repair was the method of choice where applicable as this was associated with the lowest complication rate. In our limited experience, patching of the IVC with a segment of long saphenous vein allowed adequate tumor resection without constricting the IVC. In the single case in which a primary repair was performed, tumor recurred although it is uncertain as to whether a wider resection and caval reconstitution would have changed the outcome.

When radical surgery is performed, 5- and 10-year results of 49.4% and 29.5% respectively have been reported [1]. However, recurrence rates are as high as 50% and thus surgery may simply be providing palliation in many cases [3]. Tumors of the retrohepatic IVC tend to present earlier as a result of pressure exerted on surrounding structures and as such have a superior prognosis with 5- and 10-year survival rates of 56.7% and 47.3% [1]. Other favorable factors identified by the registry include abdominal pain, the presence of a mass and the ability to achieve clear resection margins whereas poor prognostic indicators include high-grade tumors, suprahepatic tumors, and presentation with IVC occlusion or Budd-Chiari syndrome.

The role of chemotherapy and radiotherapy in the treatment of IVC leiomyosarcoma, either as a primary modality or as an adjuvant, remains to be proven. In this series, different chemotherapy protocols provided good palliation for a patient presenting with advanced disease. In the remaining three patients, only two underwent radiotherapy with a view to obtaining local control and prevention of local recurrence.

## Conclusion

Whilst IVC leiomyosarcomas are certainly rare, long-term survival depends on obtaining an early diagnosis and performing extensive surgery. The more frequent use of CT in the investigation of patients with abdominal symptoms may aid the earlier diagnosis of these tumors.

## Competing interests

The author(s) declare that they have no competing interests.

## Authors' contributions

**SA**: writing the article and literature review

**GJM**: writing the discussion and searching the literature

**DS**: operative procedure and care of one of the studied cases

**IW**: reviewing the article and operative procedure and care of one of the studied cases

**AGD**: contributing the pathological aspects of the manuscript

**MCP**: overall supervision of the article writing
